# Case of Microcephaly after Congenital Infection with Asian Lineage Zika Virus, Thailand

**DOI:** 10.3201/eid2409.180416

**Published:** 2018-09

**Authors:** Thidathip Wongsurawat, Niracha Athipanyasilp, Piroon Jenjaroenpun, Se-Ran Jun, Bualan Kaewnapan, Trudy M. Wassenaar, Nattawat Leelahakorn, Nasikarn Angkasekwinai, Wannee Kantakamalakul, David W. Ussery, Ruengpung Sutthent, Intawat Nookaew, Navin Horthongkham

**Affiliations:** University of Arkansas for Medical Sciences, Little Rock, Arkansas, USA (T. Wongsurawat, P. Jenjaroenpun, S.-R. Jun, D.W. Ussery, I. Nookaew);; Siriraj Hospital, Mahidol University, Bangkok, Thailand (N. Athipanyasilp, B. Kaewnapan, N. Leelahakorn, N. Angkasekwinai, W. Kantakamalakul, R. Sutthent, N. Horthongkham);; Molecular Microbiology and Genomics Consultants, Zotzenheim, Germany (T.M. Wassenaar)

**Keywords:** Zika virus, genome sequence, congenital Zika virus infection with microcephaly, pregnancy, viruses, vector-borne infections, Thailand, birth defects, congenital infection, microcephaly, Asian lineage

## Abstract

We sequenced the virus genomes from 3 pregnant women in Thailand with Zika virus diagnoses. All had infections with the Asian lineage. The woman infected at gestational week 9, and not those infected at weeks 20 and 24, had a fetus with microcephaly. Asian lineage Zika viruses can cause microcephaly.

Although Zika virus has circulated in Asia longer than in the Americas, only 3 confirmed cases of congenital Zika virus infection with microcephaly have been reported in Asia (2 Thailand, 1 Vietnam) (*1*). As of June 2018, the genomic sequences of the viruses from these 3 cases have not been reported; thus, whether these cases were caused by an Asian lineage or an imported American lineage is unknown.

Several mechanisms involving virus genome sequences have been proposed to explain how Zika virus might cause microcephaly (*2*). Liang et al. (*3*) showed in vitro that replication of both the African (strains MR766 and IbH30656) and American (strain H/PF/2013) lineage viruses suppress Akt phosphorylation; this suppression is caused by an accumulation of mutations in the Zika virus genome that increase the number of phosphorylation sites on virus proteins that compete with host proteins for phosphorylation. Yuan et al*.* proposed that a serine to asparagine substitution (S17N) in the premembrane protein (stably conserved in the American lineage but not in the Asian) contributes to the onset of microcephaly (*4*). An increased frequency of retinoic acid response elements in the American lineage genome versus the Asian lineage genome has also been observed (*2*). We question these explanations because we report a confirmed case of congenital Zika virus infection with microcephaly in Thailand caused by an Asian lineage virus.

We sequenced 7 Zika virus genomes obtained from 5 patients, including 3 pregnant women (PW1–3), in 2016 and 2017. PW1 had fever, maculopapular rash, and mild conjunctivitis at 24 weeks of gestation. Her urine sample was positive for Zika virus (BKK05, GenBank accession no. MG807647), and she gave birth to an infant without birth defects at full term. PW2 had a suspected Zika virus infection at 9 weeks’ gestation with high fever, maculopapular rash, and mild conjunctivitis. At 16 weeks, a sample of the amniotic fluid was positive for Zika virus (BKK03, GenBank accession no. MG548660). The pregnancy was terminated at 17 weeks. Autopsy of the fetus demonstrated a head circumference of 12.5 cm (less than the third percentile for this gestational age); Zika virus was detected in the brain (BKK02, GenBank accession no. MF996804) and placenta (BKK04, GenBank accession no. MG548661). No other etiologic agents associated with birth defects (cytomegalovirus, herpes simplex virus types 1 and 2, rubella virus, syphilis virus, *Toxoplasma gondii*, *Treponema pallidum*) were detectable by real-time PCR. PW2 had detectable hepatitis B viral surface antigen but no concurrent medical conditions. These findings suggest that Zika virus was the causative agent of this case of microcephaly. PW3 had a maculopapular rash without fever or conjunctivitis and received a Zika virus diagnosis at 20 weeks’ gestation. Her urine sample was positive for Zika virus (BKK07, GenBank accession no. MH013290), and she gave birth to a healthy infant at full term. The last 2 samples were from a 6-year-old child with mild fever and maculopapular rash (BKK06, GenBank accession no. MG807647) and a 64-year-old man with fever and maculopapular rash (BKK01, GenBank accession no. KY272987).

We retrieved 121 nonredundant Zika virus genomes (444 viruses, 99.9% nucleic acid identity cutoff) from GenBank to compare these isolates by phylogenetic analysis. All 7 BKK Zika virus isolates grouped within the Asian lineage (Figure). Virus from the amniotic fluid (BKK03), fetal brain (BKK02), and placenta (BKK04) of PW2 closely resembled each other (5 mismatches in BKK04 and 6 in BKK03, overall 99.898% identity). These 3 isolates were separated on the tree from their closest neighbor, a 2016 isolate from Singapore, by 40 mismatches. The number of retinoic acid response elements and predicted phosphorylation sites in BKK01–BKK07 was the same as the number in other Asian lineage Zika viruses (*2*). Also, the S17N substitution in premembrane was absent in all 7 isolates. Thus, all 3 proposed mechanisms failed to explain the case of congenital Zika virus infection with microcephaly in PW2. This case clinically resembled that of a woman in Finland infected during week 11 of pregnancy while traveling in Mexico, Guatemala, and Belize (*5*); in that case, Zika virus was detected in the brain of the aborted fetus at week 21.

**Figure:**
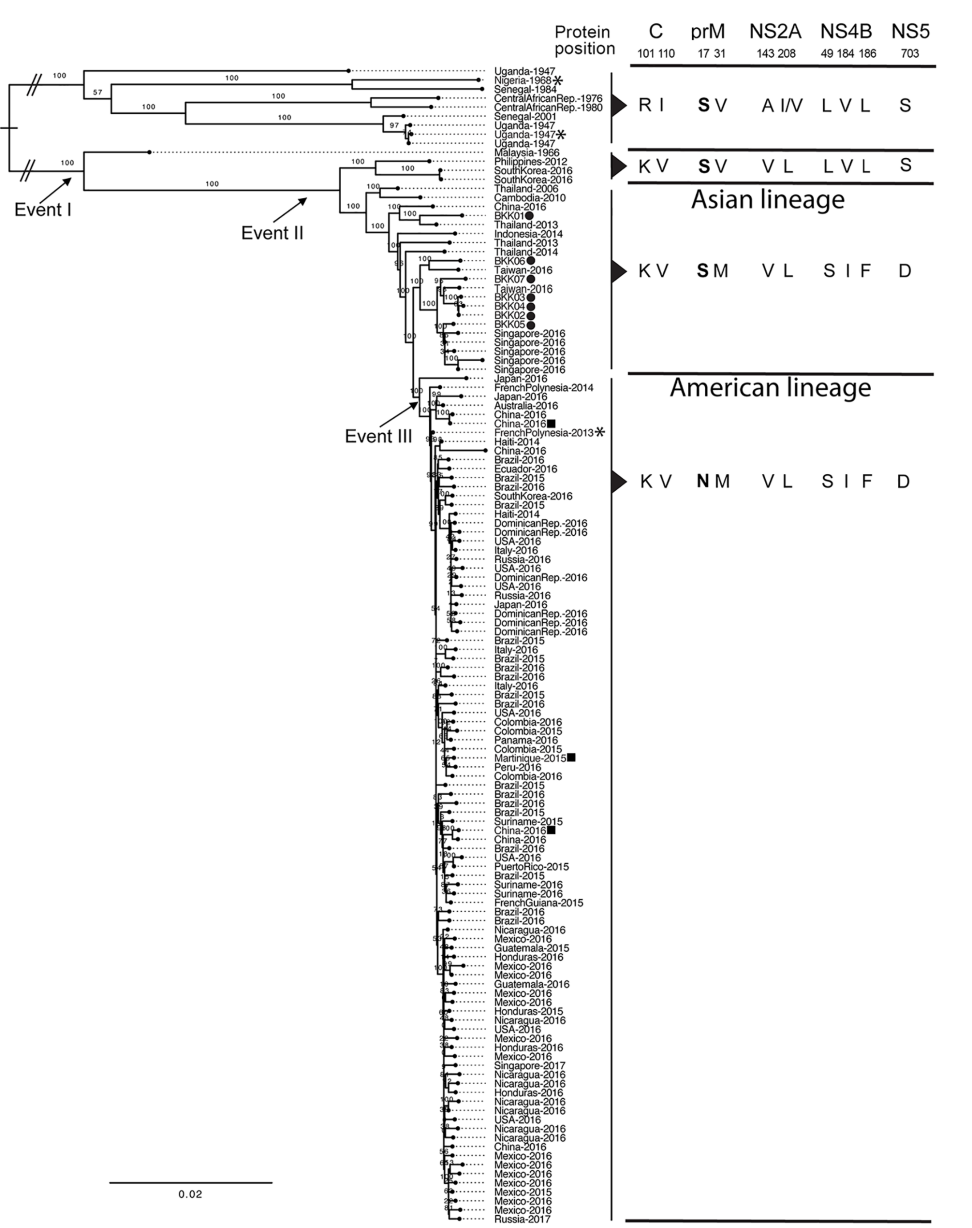
Maximum-likelihood phylogenetic analysis of nonredundant Zika virus genomes including 7 isolates from patients in Thailand, 2016–2017, and amino acid changes corresponding with 3 evolutionary events (*2*). Circles indicate the Zika virus isolates from this report; the Zika virus strains used by Liang et al. (*3*) are indicated by asterisks and Yuan et al. (*4*) by squares. The key amino acid residue changes corresponding with the 3 evolutionary events (*2*) are shown, and the conserved amino acid substitution S17N, present in the American lineage but not in the other lineages, is in bold. The amino acid residues of the 7 isolates from this report are identical to those of the other Asian lineage isolates. C, capsid; prM, premembrane; NS, nonstructural protein. Scale bar indicates nucleotide changes per basepair.

The 3 cases in pregnant women described here support the hypothesis that the timing of Zika virus infection during pregnancy might be a key contributor to the development of microcephaly during congenital Zika virus infection. PW2 was infected around week 9 of gestation, during embryonic neurulation and cortical neurogenesis, which lay the foundation for the developing brain. Infection during week 20 (for PW3) and 24 (for PW1) of gestation did not led to microcephaly. Our observations are in agreement with reports involving American lineage Zika viruses that show a high risk for microcephaly when infection occurs before week 21 (*6*), during weeks 7–14 (*7*), or during the first trimester (*8**–**10*). Our findings show that Zika viruses circulating in Asia can cause microcephaly, just like American strains.
